# Citrullination and Carbamylation in the Pathophysiology of Rheumatoid Arthritis

**DOI:** 10.3389/fimmu.2015.00192

**Published:** 2015-04-27

**Authors:** Ger J. M. Pruijn

**Affiliations:** ^1^Department of Biomolecular Chemistry, Radboud Institute for Molecular Life Sciences, Institute for Molecules and Materials, Radboud University, Nijmegen, Netherlands

**Keywords:** carbamylation, citrullination, extracellular traps, NETosis, peptidylarginine deiminase, peptidylcitrulline, peptidylhomocitrulline, rheumatoid arthritis

## Abstract

The discovery that citrullination was crucial for the recognition of antigens by the most disease-specific class of autoantibodies in rheumatoid arthritis (RA) had a huge impact on studies aimed at understanding autoimmunity in this disease. In addition to the detailed characterization of anti-citrullinated protein antibodies, various studies have addressed the identity of citrullinated antigens. These investigations were facilitated by new methods to characterize these proteins, the analysis of protein citrullination by peptidylarginine deiminases, the generation of a catalog of citrullinated proteins present in the inflamed joints of patients and the finding that the formation of extracellular traps is dependent on the activity of peptidylarginine deiminase activity. Recently, it was found that in addition to citrullination also carbamylation, which results in chemically highly related modified proteins, yields antigens that are targeted by rheumatoid arthritis patient sera. Here, all of these aspects will be discussed, culminating in current ideas about the involvement of citrullination and carbamylation in pathophysiological processes in autoimmunity, especially RA.

## Introduction

**Figure 1:**
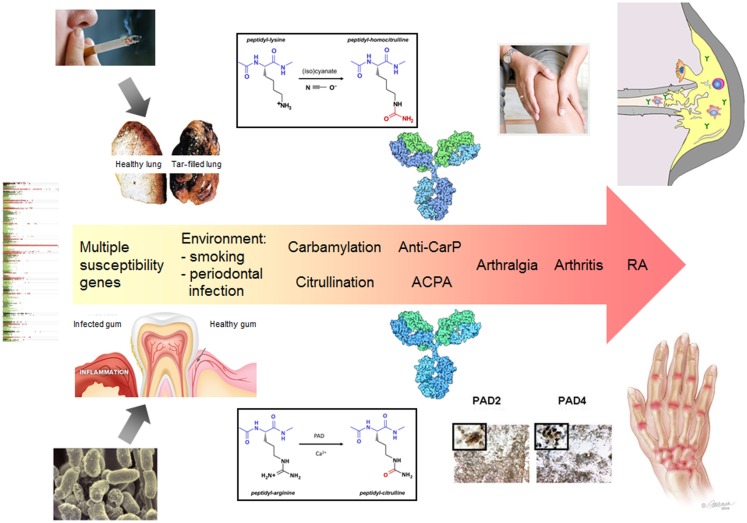
**The central role of citrullination and carbamylation in the pathology of RA**. The exposure to certain environmental factors (e.g., smoking or an infection with Porphyromonas gingivalis) leads to local protein modification by citrullination and/or carbamylation. In conjunction with an inflammatory process, this may elicit an immune response to citrullinated and/or carbamylated proteins in genetically susceptible individuals. A secondary event associated with inflammation in synovial joints results in citrullination and/or carbamylation in these tissues, e.g., as a result of NETosis, which is followed by activation of the ACPA/anti-CarP response and spreading of the autoantibody response to multiple modified epitopes, generated by PAD activation and release to the extracellular space. Certain immune complexes will boost the inflammatory process, which ultimately will result in arthritis. The continuous presence of activated PAD in the inflamed joints may also lead to the functional inactivation of extracellular matrix proteins and chemokines, which further contributes to tissue destruction. Also the autoantibodies produced may contribute to the latter process by directly activating osteoclasts.

Two forms of post-translational modification (PTM), citrullination and carbamylation, result in the generation of two non-standard amino acids in polypeptides, citrulline and homocitrulline, respectively, which are chemically highly related. Peptidylcitrulline is produced by the deimination of peptidylarginine, which is catalyzed by peptidylarginine deiminases (PADs), a family of structurally related enzymes that show distinct tissue-specific expression patterns (1). Peptidylhomocitrulline is the product of the reaction of cyanate (OCN)^-^ with peptidyllysine. The side chains of both citrulline and homocitrulline are characterized by the presence of an ureido group (-NH-CO-NH_2_); the difference is the length of the side chains, which is one methylene longer in homocitrulline (Figure 1).

Citrullination has been reported to be associated with a variety of diseases, including cancer, neurodegenerative diseases, and autoimmune diseases (1). Carbamylation has been implicated in atherosclerosis, kidney disease, and inflammation (2). A special case is rheumatoid arthritis (RA), in which a frequent humoral immune response to proteins containing (homo)citrulline was observed. The resulting autoantibodies to citrullinated proteins (ACPA) are considered to represent the most specific biomarker for RA and the presence of these autoantibodies in patient sera has been added to the criteria for the classification of RA in 2010. The more recently identified anti-carbamylated protein (anti-CarP) autoantibodies also show specificity for RA and, at least in part, appeared to overlap with ACPA reactivities, although also anti-CarP-positive, ACPA-negative patients have been described.

Citrullination has been reported to be involved in hair growth, skin keratinization, myelin formation, the regulation of gene expression, extracellular trap formation, and several other processes [reviewed in Ref. (1)]. It is not yet known whether carbamylation has physiological functions. Conditions that enhance cyanate levels, such as uremia, lead to the accumulation of carbamylated proteins.

There is increasing evidence for a pathophysiological role of protein citrullination and carbamylation, and of the autoantibodies to these proteins in RA. The elucidation of the corresponding molecular mechanisms is complicated due to the heterogeneity of citrullinated and carbamylated proteins and the heterogeneity of the autoantibodies to these proteins. To better understand these processes, high-throughput tools and techniques are required to characterize the “citrullinome” and “carbamylome”, both in health and disease, and to obtain more insight in the spectrum of ACPA and anti-CarP specificities. In this article, I will briefly discuss new technologies for the detection of such proteins, address the substrate specificity of the PAD enzymes, and describe studies aimed at the identification of citrullinated and carbamylated proteins in diseased tissues of RA. Furthermore, I will address the questions how tolerance to citrullinated and carbamylated antigens breaks and autoantibody production begins and I will discuss the (potential) role of these forms of protein modification in pathophysiological processes.

## Methods to Detect Citrullinated and Carbamylated Proteins

The ability to identify and quantify citrullinated and carbamylated proteins is key to understanding the role of these PTMs in physiological and pathophysiological processes. The specific detection of peptidyl(homo)citrulline in complex biological samples is challenging due to the very small chemical difference between citrulline and arginine on the one hand and homocitrulline and lysine on the other hand. Moreover, with many methods the discrimination between citrullinated and homocitrullinated proteins is hard or not possible. The most widely applied method for peptidylcitrulline is the so-called anti-modified citrulline approach, originally developed by Dr. Tatsuo Senshu in Japan (3). In this method, the proteins are incubated with compounds that are specifically reactive with the ureido side chain, resulting in an adduct that can be detected with anti-modified citrulline antibodies. Many results obtained with this elegant approach, however, have to be re-evaluated, because this method will detect carbamylated proteins as efficiently as citrullinated proteins and there is increasing evidence that carbamylation occurs under (patho)physiological conditions (4).

Unfortunately, several attempts to elicit new anti-modified citrulline antibodies in rabbits failed. To some extent, the lack of these antibodies could be solved by using antibodies that target the citrullinated isoforms of specific proteins, e.g., anti-citrullinated fibrinogen and anti-citrullinated chemokine antibodies. For studies that are aimed at the global detection of citrullinated or carbamylated proteins such antibodies are obviously not suitable, but recently monoclonal anti-modified citrulline antibodies have been generated, which can be applied in the procedure originally developed by Dr. Senshu.

Another method that was recently described to be applicable for the detection of (homo)citrulline containing proteins is based on the chemical reaction of the ureido group with phenylglyoxal under highly acidic conditions. Methods to detect citrullinated/carbamylated proteins in complex samples based on this compound, however, appeared to be hampered by relatively high background reactivities (4).

Finally, the analysis of proteins by mass spectrometry provides very attractive possibilities to identify and characterize citrullinated and carbamylated proteins. For more information on the applicability of these methods, I refer the reader to several recent review articles (4, 5). Compared to other techniques, one of the main advantages of mass spectrometry is that it discriminates between citrullination (mass increase 0.98 Da) and carbamylation (mass increase 43.02 Da) and that by tandem mass spectrometry also the modification site can be mapped. We have used mass spectrometric approaches to get more information on the substrate specificity of PAD enzymes and to characterize citrullinated proteins in synovial fluid samples of RA patients, which will be addressed in the following paragraphs.

## Specificity of Citrullination and Carbamylation (PAD; MPO)

Since peptidylcitrulline is generated by an enzymatic modification and peptidylhomocitrulline results from a chemical modification reaction, the specificity of citrullination will generally be higher than that of carbamylation. Several studies have addressed the substrate requirements for PAD enzymes. Although both favored and disfavored amino acids at positions flanking the deiminated arginine have been identified, the results demonstrated that it is difficult to derive a consensus sequence for citrullination sites. Nevertheless, for the four amino acids flanking the hPAD4 citrullination site, two on each side, the proposed consensus sequence is (M/K)–(D/S)–R–(G/D)–(H/W). The results also demonstrated that the human PAD4 displays a more pronounced substrate specificity than the human PAD2 (6).

Unlike citrullination, carbamylation is a chemical modification, which is mainly induced by the presence of cyanate. The formation of cyanate may result in a reaction with any accessible primary amine, including that of the lysine side chain and the amine at the N-terminus of polypeptides. Carbamylation can be stimulated by myeloperoxidase (MPO), which is most abundantly expressed in neutrophils, and which can convert thiocyanate coming from cigarette smoke or dietary compounds into (iso)cyanate in the presence of hydrogen peroxide. Also the spontaneous degradation of urea, which is constantly and ubiquitously generated in the body, results in the formation of cyanate (2). Although under normal physiological circumstances, cyanate levels will be too low to induce substantial carbamylation, conditions like uremia, inflammation, and the exposure to cigarette smoke enhance cyanate levels.

## Citrullinated Proteins in RA

In view of the putative pathophysiologic role of citrullinated protein-specific immune complexes (see below) in RA, it is important to obtain a comprehensive view of the citrullinated proteins present in the inflamed joints of patients with RA. A systematic analysis of citrullinated proteins present in the synovial fluid of RA patients by mass spectrometry led to the identification of 53 polypeptides containing one or more citrulline residues, which comprises 28% of all proteins identified (7). A comparison of the data obtained with material from different patients indicates that this is probably only a minority of the citrullinated proteins occurring in the inflamed joints of RA patients, implying that many proteins will be citrullinated in these tissues. Among the proteins that were found to be citrullinated were fibrinogen, vimentin, fibronectin, and histones, proteins that were reported to be modified in inflamed tissues in previous studies. When the nature and frequency of amino acids flanking the citrulline were compared with the substrate specificity data described above, this suggested that citrullination in the RA synovium is exerted by a combination of PAD enzymes, e.g., PAD2 and PAD4. Indeed, both PAD2 and PAD4 have been reported to be expressed in the inflamed synovium of RA patients, in contrast to the other PAD isotypes (8).

Currently, hardly anything is known about the identity of carbamylated proteins that may play a role in autoimmunity. However, because *in vivo* carbamylation is mainly dependent on the activity of MPO (located in granules of neutrophils) and hydrogen peroxide (generated by activated neutrophils), many proteins are also expected to become carbamylated in the inflamed joints.

## Extracellular Traps

During inflammation, e.g., caused by infections with bacteria, yeast, or viruses, neutrophils initiate a programed cell death termed NETosis. This process results in the release of unwound chromatin from the cells, termed neutrophil extracellular traps (NETs). Also other inflammatory cells have been demonstrated to produce extracellular traps upon activation by pro-inflammatory stimuli. Most interestingly, PAD4 activity has been found to be required for NETosis and citrullinated histones are incorporated into NETs (9). It is likely that activated PAD4 is also released from the neutrophils during NETosis and this may lead to the citrullination of additional proteins in the extracellular space. Also, deficiencies in neutrophil MPO result in impaired release of NET chromatin. In addition to the association with citrullinated histones, extracellular traps are decorated with anti-microbial compounds originating from neutrophil granules, including MPO (10). Thus, extracellular traps represent macromolecular assemblies, which are potentially associated with both citrullination and carbamylation and therefore it is tempting to speculate that they play a role in the initiation of the (homo)citrulline-specific immune response in RA or in the progression of this response. Indeed, autoantibodies to citrullinated histones have been detected in RA patients, patients with systemic lupus erythematosus (SLE) and patients with Felty’s syndrome (FS).

## Citrullination and Carbamylation in Pathophysiological Processes

The first autoimmune disease in which citrullination was suggested to play a role was multiple sclerosis (MS). Myelin basic protein (MBP) is one of the proteins that is citrullinated under normal physiologic conditions, but in MS hypercitrullination of this protein was observed, and the resulting MBP isoforms differentially react with T-cells from MS patients (11).

Several studies have demonstrated that the humoral immune response to citrullinated (and carbamylated) proteins in RA is not merely an epiphenomenon. A pathophysiologic role is supported by the very early induction of ACPA and anti-CarP antibodies in RA, often long before the disease becomes clinically manifested, the more severe progression of the disease in seropositive patients, the induction of arthritis in animal models upon immunization with citrullinated antigens, and the exacerbation of arthritis by ACPA in murine models of arthritis (12).

As a consequence of the very early generation of ACPA and anti-CarP antibodies it is difficult to obtain information on the factors triggering the immune response to these modified proteins. Nevertheless, it is well known that genetic and environmental factors play an important role (Figure 1). Smoking (cigarette) has been demonstrated to be a prominent risk factor and genome-wide analyses have identified multiple genes that are associated with the development of RA (genome-wide association studies have identified more than 100 risk loci). Another environmental risk factor is the periodontal pathogen *Porphyromonas gingivalis*, which expresses an enzyme with peptidylarginine deiminase activity. It has been hypothesized that in genetically susceptible individuals an inflammation in the lungs or the oral cavity may lead to autoantigen citrullination that triggers the generation of ACPA or anti-CarP antibodies (13, 14). Although this will not immediately lead to arthritis, a secondary event that is associated with joint inflammation may activate the (homo)citrulline-specific B-cells and subsequently the immune response may spread to other citrullinated/carbamylated epitopes generated in the inflamed joints. It is interesting to note that smoking results in elevated thiocyanate levels in the lungs, which can be converted by MPO into cyanate in the presence of hydrogen peroxide. Therefore, in a subset of RA patients, the ACPA/anti-CarP response may have been triggered by carbamylated autoantigens in the lungs. Alternatively, in the case of a *P. gingivalis* infection, autoantigen citrullination may be catalyzed by either activated PAD from neutrophils or by the bacterial PAD.

Several studies show that the recognition of citrullinated autoantigens is not restricted to B cells. In the context of a proper genetic background (HLA-DRB1 shared epitope), also T cells may be activated by citrullinated epitopes and play a role in the etiology of RA. RA patients recently were reported to have significantly higher frequencies of peripheral citrulline-specific T cells than healthy subjects (15).

The role of NET-associated citrullination and carbamylation in these processes remains to be established. As already mentioned above, prominent NET autoreactivity has been detected in the sera from patients with RA, SLE, and FS. Autoantibodies to citrullinated histones appeared to be produced in the majority of FS patients and in a subset of RA and SLE patients (9). It is still an open question whether these reactivities are due to ACPA and/or anti-CarP antibodies that are cross reactive with citrullinated epitopes of histones or whether these antibodies were elicited by citrullinated histones. Additional evidence for a functional relationship between NETosis and autoimmunity came from experiments in which circulating (RA, SLE) and synovial fluid (RA) neutrophils from autoimmune patients were shown to display enhanced NETosis compared to neutrophils from healthy controls and from osteoarthritis patients, respectively. Moreover, RA sera and immunoglobulin fractions from RA patients which high levels of ACPA and/or rheumatoid factor significantly enhance NETosis (16). Finally, SLE sera were reported to have a reduced capacity to degrade NETs. Taken together, these data indicate that autoimmunity, at least in RA and SLE, is associated with changes in NET formation and/or degradation, but the involvement of NET-associated citrullinated and carbamylated proteins is yet unknown.

Direct effects of citrullination in pathophysiological processes, especially in RA, may also originate from the citrullination of extracellular matrix proteins, like fibronectin and collagen II, which affects integrin-mediated cell adhesion (17). Citrullination has also been reported to affect the activity of chemokines, which may dysregulate the network in which these immunomodulators function, leading to uncontrolled inflammation (18).

In view of the limited specificity of carbamylation, it is likely that the function of multiple proteins is affected by this type of modification under conditions associated with elevated levels of cyanate. However, autoimmunity-related pathophysiological processes directly mediated by the carbamylation of proteins involved, have not been identified yet.

## Concluding Remarks

Citrullination and carbamylation have been demonstrated to represent PTM that are important for the generation of an immune response in several autoimmune diseases, particularly RA. Increasing evidence points to a role of the modified proteins and the immune response mounted against these proteins in pathophysiological processes in these diseases. However, it is important to note that citrullination and, to a lesser extent, carbamylation have normal physiological functions and the differentiation between physiological and pathological citrullination and carbamylation remains to be defined, both qualitatively and quantitatively.

Also the pathologic stimuli of citrullination and carbamylation are still largely unknown, although there are strong indications that in the case of RA modification of proteins in the lung and/or the gums induced by environmental factors may initiate an immune response. Moreover, a role for extracellular traps and the release of enzymes and molecules from the cells during extracellular trap formation has been suggested. In the inflamed joints of RA patients, many proteins are citrullinated and this modification can have consequences for the biochemical function of these proteins, as well as for the immune response and immune complex formation. It remains an open question which citrullinated epitopes are most relevant for pathophysiological processes.

The disease-specificity of the ACPA and anti-CarP immune responses, as well as the evidence for the involvement of the corresponding protein modifications and these antibodies in pathophysiological processes, strongly suggest that modulation of citrullination and/or carbamylation might be a potential therapeutic approach to treat patients with RA and other autoimmune diseases associated with these modifications. Therefore, future studies should be aimed at defining the most relevant (homo)citrullinated epitopes, determination of the enzyme isotype(s), and environmental factors that play the most important role, and the generation of molecules that can interfere with the molecular interactions and conversions involved in a highly specific manner.

## Conflict of Interest Statement

The author is co-founder and shareholder of ModiQuest B.V., a company focused on the development of novel monoclonal antibody therapeutics and diagnostics for autoimmune and oncology targets, with a special interest in citrullination, including the anti-CCP test.
